# Changes in cerebral function parameters in HIV-1 infected subjects undergoing a treatment simplification to darunavir/ritonavir

**DOI:** 10.1186/1758-2652-13-S4-P52

**Published:** 2010-11-08

**Authors:** LJ Garvey, C Higgs, P Mohammed, M Nelson, A Winston

**Affiliations:** 1 Imperial College, HIV Medicine, London, UK; 2 Chelsea and Westminster, HIV Medicine, London, UK; 3 Janssen Cilag, Virology, Buckinghamshire, UK

## Purpose of study

Concerns exist regarding the central nervous system (CNS) penetration and cognitive sequelae when utilising novel, nucleoside-sparing treatment strategies such as protease-inhibitor monotherapy.

## Methods

The aim of this study was to assess changes to cerebral function parameters in HIV-1 infected subjects, stable on antiretroviral therapy with a plasma HIV RNA<50 copies/mL, randomised to commence on a one to one basis, either darunavir/ritonavir (800/100 mg one daily) alone (DRVmono) or with nucleoside analogues (DRVnrti), within the MONET study. Cerebral function was assessed via a detailed, validated, computerised neurocognitive function assessment (CogState™) and cerebral metabolites measured via cerebral proton spectroscopy in three anatomical locations (frontal grey and white matter and right basal ganglia (RBG)), at baseline and after 48 weeks. Associations between cerebral function parameters and study treatment arms were evaluated using linear regression.

## Results

6 subjects were enrolled (3 assigned to each treatment arm) with a mean age of 44 years (SD 4.5) and 83% male. Mean baseline plasma CD4+ cell count was 503 cells/uL (SD 173). Over 48 weeks, mean score improvements were observed in 6 of 8 neurocognitive tasks assessed including speed domains (2.5% increase identification speed), accuracy domains (4.6% increase non-visual learning) and executive function (30% reduction in errors). Reductions in cerebral metabolite markers of cerebral inflammation (choline:creatine (Cr) and myo-inositol:Cr ratios) were also observed in all cerebral locations assessed (maximum reduction of 28% myo-inositol:Cr ratio in frontal grey matter). No associations between study treatment arm and these improvements in cerebral function parameters were observed (p>0.06 all values).

## Conclusion

On detailed assessment of cerebral function, overall improvements were observed over 48 weeks in subjects allocated to either DRVmono or DRVnrti. Despite very small numbers, our study highlights future tools which can practically be utilised to assess cerebral function in HIV-treatment programmes.

